# Male breast cancer: A closer look at patient and tumor characteristics and factors associated with survival

**DOI:** 10.1111/1759-7714.13611

**Published:** 2020-09-15

**Authors:** Jing Zhao, Bin Wang, Jing Zhao, Yiran Mao, Jun Liu, Yanfang Yang

**Affiliations:** ^1^ Second Department of Breast Surgery Tianjin Medical University Cancer Institute and Hospital Tianjin China; ^2^ National Clinical Research Center for Cancer, Key Laboratory of Cancer Prevention and Therapy Tianjin China; ^3^ Tianjin's Clinical Research Center for Cancer, Key Laboratory of Breast Cancer Preventionand Therapy Tianjin Medical University Tianjin China; ^4^ Department of Ultrasound Diagnosis Tianjin Medical University Cancer Institute and Hospital Tianjin China

**Keywords:** Clinical characteristic, male breast cancer, prognosis, therapy

## Abstract

**Background:**

The prognostic effect of molecular subtypes on male breast cancer (MBC) remains unclear. The aim of this study was to evaluate the clinicopathological and prognostic factors of MBC patients.

**Methods:**

From 1 January 1990 to 31 December 2014, the data of 152 MBC and 304 female breast cancer (FBC) patients were identified and extensively compared.

**Results:**

Compared with the FBC group, MBC patients were found to have a higher rate of cancer family history (30.9% vs. 18.4%, *P* = 0.001), mass around the areola area (37.5% vs. 5.6%, *P* = 0.000), lymph node invasion (44.1% vs. 34.2%, *P* = 0.006) and hormonal receptor positivity (66.4% vs. 49.3%, *P* = 0.027). Luminal A was the most common subtype accounting for 69.8%, whereas HER2‐positive (12.7%) and TNBC (1.6%) subtypes were rare in the MBC group. However, it was significantly lower for MBC than for FBC who received endocrine therapy (38.8% vs*.* 49.3%, *P* = 0.041). MBC showed the worse overall survival (OS) and disease‐free survival (DFS) than those of FBC patients. However, 10‐year OS and DFS were similar between MBC and FBC patients in the subgroups of nonluminal subtype (*P* < 0.001), but worse in MBC patients than those in FBC patients in the subgroups of luminal A (*P* = 0.004 for OS; *P* = 0.002 for DFS) and luminal B (*P* = 0.006 for OS; *P* = 0.003 for DFS). Multivariate analysis indicated tumor size, radical mastectomy and endocrine therapy as independent risk factors for OS and DFS of MBC patients.

**Conclusions:**

Our study determined that MBC patients possessed a worse prognosis, usually with lymph node invasion, and were estrogen receptor (ER), progesterone receptor (PR)‐positive and human epidermal growth factor receptor (HER2)‐negative. Molecular subtypes based on FBC did not provide the same prognostic information in MBC, even in the luminal groups.

## Graphical abstract

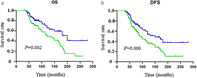

In our study we compared the similarities and differences between male and female breast cancer. The biological characteristics and prognostic factors of male breast cancer were analyzed.

## Introduction

Male breast cancer (MBC) is a rare disease accounting for less than.

1% of all breast cancer.^1^ The lifetime risk of breast cancer is about 1:1000 for a man, whereas it is approximately 1:8 for a woman in the United States.^2^ Due to its rarity, few prospective clinical trials focusing on MBC have been performed and many of the management approaches used for MBC are based on those used for women.^3^ However, there are substantial differences between male and female breast cancer (FBC).^4^, ^5^

The most outstanding distinctive features in MBC patients are elevated positive expressions of estrogen receptor (ER) and progesterone receptor (PR), but rarely positive expression of human epidermal growth factor receptor 2 (HER2).^6^, ^7^, ^8^ Meanwhile, MBC patients have been reported to present at an older age,^7^ with more frequent lymph node metastases and higher nodal stage than those of FBC patients. Additionally, risk factors for MBC patients have also been reported to be different in that they are more likely to carry a BRCA2 mutation rather than the BRCA1 mutation found in FBC patients.^9^

Over the past decade, the molecular subtypes generated by microarray‐based gene expression studies have elucidated the breast cancer heterogeneity differently.^10^ Sorlie *et al*. investigated 115 breast cancers from females to determine the expression of 534 intrinsic genes by hierarchical clustering,^11^ from which four major groups were confirmed: luminal A (43%), luminal B (20%), HER2 (10%) and TNBC (27%). In clinical practice, immunohistochemistry (IHC) is commonly used for detection of those four groups based on the expression of ER, PR, and HER2: luminal A (ER‐ or PR‐positive, HER2‐negative), luminal B (ER‐ or PR‐positive, HER2‐positive), HER2‐overexpressing (ER and PR‐negative, HER2‐positive), and triple‐negative breast cancer (TNBC: ER, PR, and HER2 all negative).^12^ This classification contributes to the treatment guidelines for breast cancer and shows great prognostic significance in FBC.^11^ However, the efficiency of the classification in MBC patients remains unclear, and the outcome of MBC patients compared with those of FBC patients under the same molecular subtypes remain unknown. In this study, we attempted to establish the clinicopathological characteristics of MBC patients compared with those of FBC patients in China. Based on a large database with long‐term follow‐up, we aimed to explain the difference in outcomes between MBC and FBC patients, particularly in those under the same subtypes, as defined by ER, PR, and HER2 expressions.

## Methods

### Patients

A total of 27 618 primary breast cancer patients underwent surgeries in the Tianjin Medical University Cancer Institute and Hospital between 1 January 1990 and 31 December 2014. Each patient in the study accepted the informed consent, and the study was approved by the ethics committee of Tianjin Medical University Cancer Institute and Hospital. A total of 172 cases were diagnosed with breast cancer in males, which accounted for 0.62% of all the breast cancer patients. Cases were excluded if they met the following exclusion criteria: clinical data and pathological data after surgery was incomplete, or standard four to six cycles of neoadjuvant chemotherapy or endocrine therapy were applied. Finally, we enrolled 152 MBC cases, while 304 FBC cases were paired as the control group in 1:2 ratio by systematic sampling with paired data methods. The matching criteria between the two groups were as follows: (i) the age difference was less than five years; (ii) the diagnosis time was less than one year; (iii) the clinical stage was the same; and (iv) if more than two FBC cases met the above matching conditions, two cases who presented the closest diagnosis time to the MBC patient were chosen.

### Clinicopathological assessment

Clinical and pathological staging was performed in accordance with the sixth edition of the American Joint Committee on Cancer TNM classification principle.^13^ Patients with stage IV disease usually received systemic therapy instead of surgery and were excluded from this study. Histologic grade of tumor was based on the criteria of Elston *et al*.^14^ ER and PR positivity was defined as at least 15% nuclear staining by IHC. HER2 expression was assessed by IHC or fluorescence in situ hybridization (FISH). HER2 was considered positive if the IHC score was +++ and negative if the score was 0 or + based on staining intensity. If the score was ++, then a further assay with FISH was performed. For subgroup analysis, the IHC classifications were as follows: luminal A: ER‐ or PR‐positive, and HER2‐negative; luminal B: ER‐ or PR‐positive, and HER2‐positive; HER2‐overexpressing: ER and PR‐negative, but HER2‐positive; TNBC: ER, PR, and HER2 were all negative.

### Statistical analysis

Differences in the characteristics were analyzed by chi‐square test for distribution and Mann‐Whitney U test for the means between groups. To investigate the difference of outcomes between MBC and FBC, we conducted survival analysis using the primary endpoints of overall survival (OS) and disease‐free survival (DFS). OS was defined as the length of time from the first diagnosis of primary breast cancer to death from any cause. DFS was defined as the length of time from the initial diagnosis to local recurrence or distant metastasis. Kaplan‐Meier product limit method was used to obtain the survival curves; log‐rank test was performed to investigate the difference in survival between groups. Multivariate Cox proportional‐hazards regression analysis was used to assess the independent prognostic significance of various pathological features on each of the previous outcomes. SPSS 17.0 software was used for statistical analysis. Values of *P* < 0.05 were considered statistically significant.

## Results

### Comparison of clinicopathological characteristics between MBC and FBC patients

Table 1 summarizes the personal features and clinical findings. MBC occurred more often in people aged >65 years as well as 10 years later than FBC patients^5^, ^7^; however, in our patient cohort, MBC patients were seemingly at a younger median age of 58.6 years without a clear‐cut distinction between FBC patients (58.6 vs. 57.9, *P* = 0.107). The proportion of positive malignant tumor family history was much higher in MBC patients than that in FBC patients (30.9 vs 18.4%, *P* = 0.001). Significantly, most MBC patients presented with an areola area mass or lesion (37.5 vs. 5.6%, *P* = 0.000). The common invasive ductal carcinoma (IDC) tumor type found on histology was higher in MBC patients than in the FBC group (92.1 vs. 72.4%, *P* = 0.407), but the difference was insignificant.

**Table 1:** Clinical characteristics of male breast cancer (MBC) and female breast cancer (FBC)

	No. of patient (%)		
Clinical parameter	MBC	FBC	*P*‐value
Mean age (years)	58.6 ± 9.7	57.9 ± 10.3	0.107
Median age (years)	59 (26–83)	57 (21–82)	
Malignancy family history			0.001*
Negative	105 (69.1)	248 (81.6)	
Positive	47 (30.9)	56 (18.4)	
Breast family history			0.113
Negative	126 (82.9)	253 (83.8)	
Positive	26 (17.1)	49 (16.2)	
Mass location			0.000 *
Areola area	57 (37.5)	17 (5.6)	
Nonareola area	95 (62.5)	287 (94.4)	
Histology			0.563
Invasive ductal carcinoma	140 (92.1%)	220 (72.4%)	
Others	12 (7.9%)	84 (27.6%)	
Clinical stage			0.858
I	31 (20.4)	62 (20.4)	
II	77 (50.7)	154 (50.7)	
III	44 (28.9)	88 (28.9)	
Surgery			0.503
RM^a^	129 (84.9)	243 (79.9)	
Non‐RM^b^	23 (15.1)	61 (20.1)	
Radiotherapy			0.932
Negative	90 (59.2)	181 (59.5)	
Positive	62 (40.8)	123 (40.5)	
Chemotherapy			0.871
Negative	40 (26.3)	66 (21.7)	
Positive	112 (73.7)	238 (78.3)	
Endocrine therapy			0.041*
Negative	93 (61.2)	154 (50.7)	
Tamoxifen	57 (37.5)	97 (31.9)	
Aromatase inhibitor	2 (1.3)	53 (17.4)	0.003*

^a^ Radical mastectomy.

^b^ Nonradical mastectomy

* *P* < 0.05.

Radical mastectomy (RM) was mostly performed in our cohort, 84.9% in MBC patients and 79.9% in FBC patients, respectively, whereas mastectomy and breast‐conserving surgery (referred to as nonradical mastectomy [non‐RM]) were used less in both the MBC and FBC groups. Chemotherapy was performed in 73.7% and radiation therapy in 40.8% of MBC patients, similar to that used in the FBC group. Among patients with ER + tumors, 38.8% of MBC patients received adjuvant endocrine therapy compared with 49.3% of FBC patients. The rate of endocrine therapy in MBC patients was significantly lower than that in the FBC group (38.8 vs. 49.3%, *P* = 0.041). Moreover, there was a dramatic difference in that less few aromatase inhibitors were used in MBC patients than were used in FBC patients (1.3 vs. 17.4%, *P* = 0.003). None of the patients were treated with Herceptin targeting HER2 amplification in our cohort.

As shown in Table 2, the mean diameters of tumors between the two groups were similar, although an insignificantly higher rate of tumor size >5 cm was observed in FBC patients (17.4 vs 13.2%, *P* = 1.141). Significantly more patients in the MBC group had lymph node invasion (44.1 vs. 34.2%, *P* = 0.046). However, no significant difference existed in the pathological stage distribution (*P* = 0.276). We used a stricter standard for the definition of ER and PR positivity than that of other studies, in which 15% nucleus staining was the dividing line. However, we still found a significantly higher proportion of ER‐positive patients in the MBC group than that in the FBC group (66.4 vs. 49.3%, *P* = 0.027). We also observed a lower rate of HER2‐positive expression in MBC patients compared with FBC patients, but the difference was without significance (11.9 vs. 16.4%, *P* = 0.304). Given the different ER/PR and HER2 status, a significant difference was found in the distribution of subgroups between the two groups. In comparison with FBCs, most luminal A (69.8%), fewer luminal B (15.9%), HER2 overexpression (12.7%), and rare TNBC cases (1.6%) were observed in the MBC group (*P* = 0.025).

**Table 2:** Pathological characteristics of male breast cancer (MBC) and female breast cancer (FBC)

	No.of patient (%)		
Pathological parameter	MBC	FBC	*P*‐value
Mean tumor diameter (cm)	3.05 ± 0.091	2.54 ± 0.043	0.441
Tumor size			1.141
≤ 5 cm	132 (86.8)	251 (82.6)	
> 5 cm	20 (13.2)	53 (17.4)	
Lymph node invasion			0.006 *
Negative	85 (55.9)	200 (65.8)	
Positive	67 (44.1)	104 (34.2)	
Histologic grade			0.276
I	30 (19.7)	75 (24.7)	
II	100 (65.8)	195 (64.1)	
III	22 (14.5)	34 (11.2)	
ER/PR status			0.027*
Negative	20 (13.2)	101 ( (33.3)	
Positive	101 (66.4)	150 (49.3)	
Unknown	31 (20.4)	53 (17.4)	
HER2 status			0.51
Negative	45 (29.6)	94 (30.9)	
Positive	18 (11.8)	50 (16.5)	
Unknown	89 (58.6)	160 (52.6)	
Molecular subtype			0.005*
Luminal A	44 (69.8)	69 (47.9)	
Luminal B	10 (15.9)	31 (21.5)	
HER2 overexpression	8 (12.7)	29 (20.1)	
TNBC	1 (1.6)	15 (10.5)	

* *P* < 0.05.

### Survival analysis

**Figure 1:**
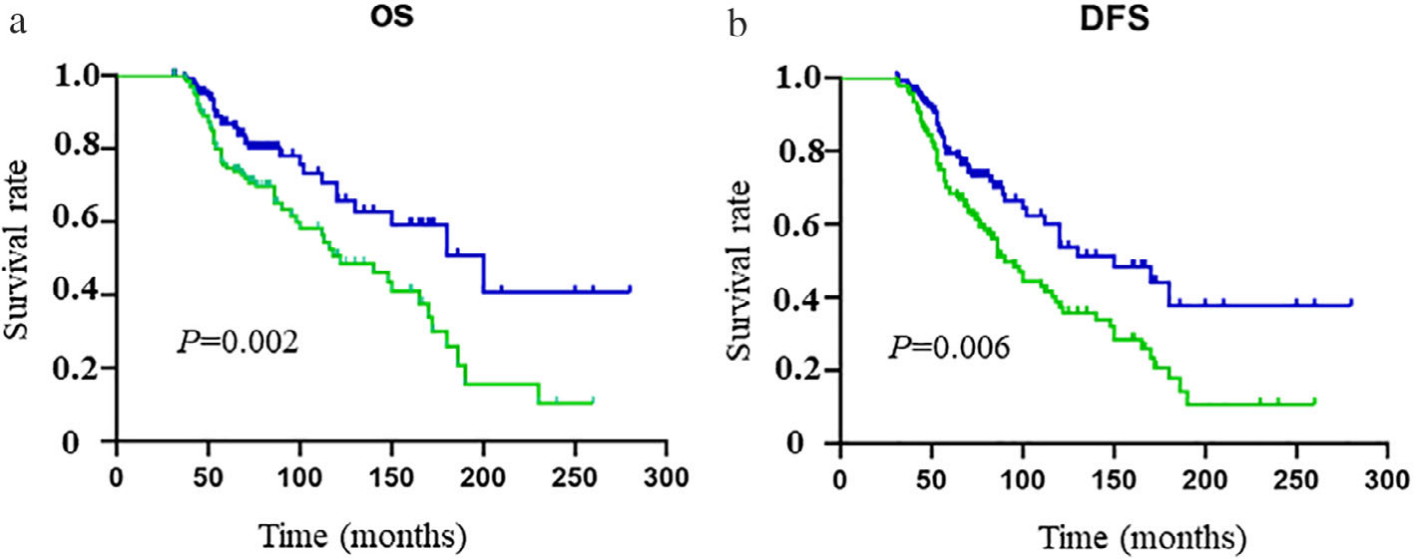
Comparison of overall outcome between MBC and FBC patients. (**a**) Overall survival (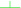) MBC, and (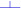) FBC; and (**b**) disease‐free survival (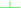) MBC, and (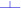) FBC.

The median follow‐up time was 85.8 months (ranging from 9 to 298 months) for the MBC group and 90.2 months for the FBC group. No significant differences were observed in the rate of local recurrence (10.3 vs. 9.7%, *P* = 0.835) between the two groups, whereas a higher rate of distant metastasis existed in the MBC group than in the FBC group (47.7 vs. 37.6%, *P* = 0.043). We also found a significantly higher rate of secondary tumor in organs other than the breast in MBC patients than in FBC patients (16.8 vs. 5.4%, *P* = 0.027). We used OS and DFS to assess patient outcomes. The five‐ and 10‐year OS of MBC were 74.6% and 50.6%, while those of FBC were 86.9% and 65.7% (all *P* < 0.05). In addition, worse five‐ and 10‐year DFS were obtained in the MBC group than those in the FBC group (68.4% vs. 79.3% and 39.8% vs. 54.3%; all *P* < 0.05). The survival curves are shown in Fig 1.

**Figure 2:**
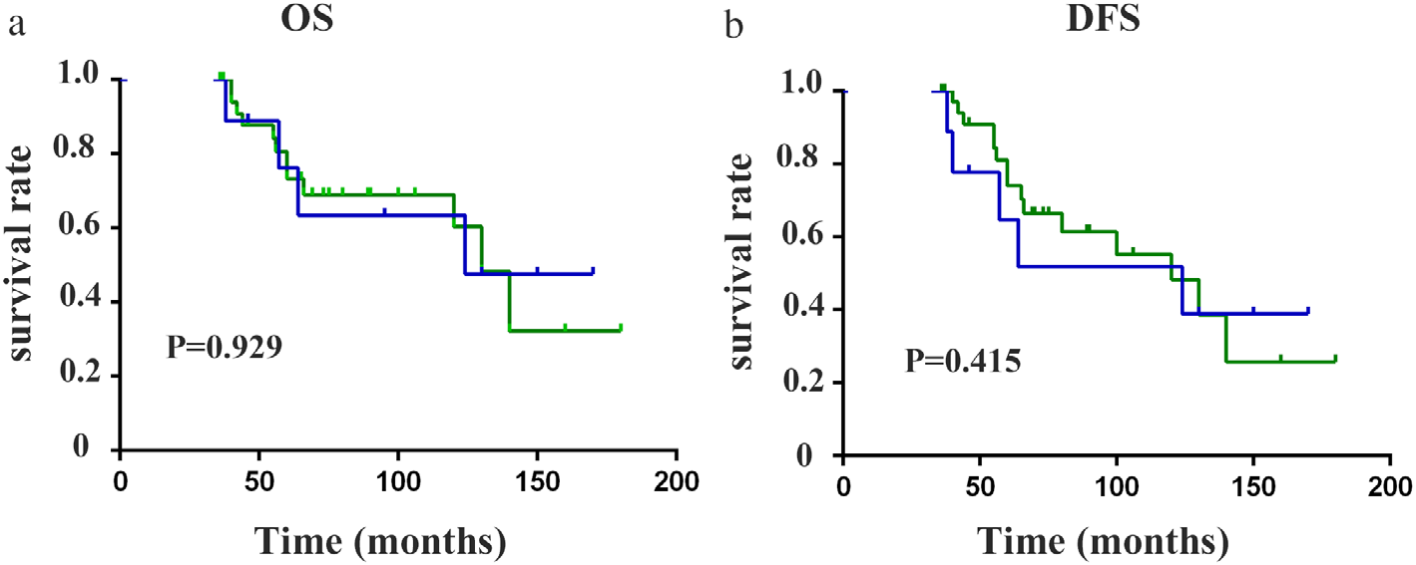
Comparison of outcome between MBC and FBC patients within a matched nonluminal subtype. (**a**) Overall survival (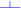) MBC, and (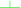) FBC; and (**b**) disease‐free survival (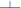) MBC, and (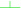) FBC.

**Figure 3:**
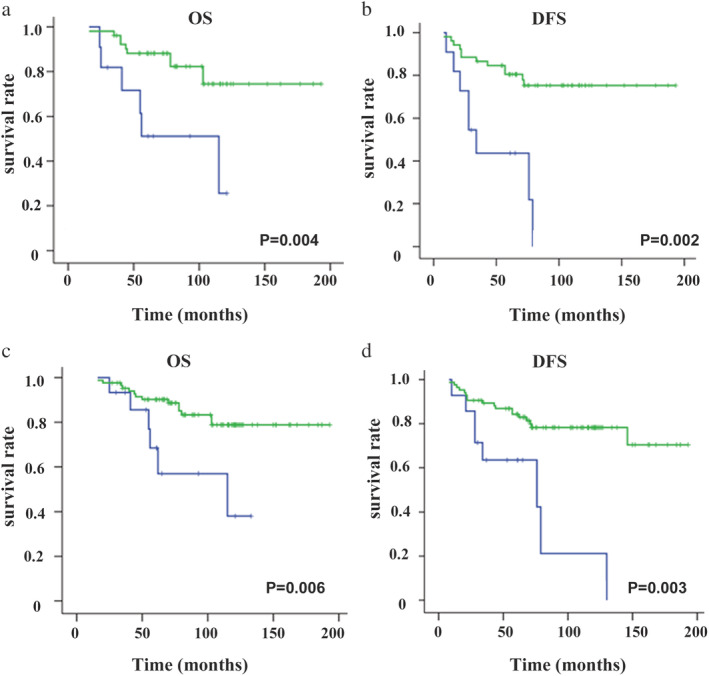
Comparison of outcome between MBC and FBC patients within a molecular subtype. Overall survival (OS) and disease‐free survival (DFS). (**a**) Luminal A group (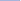) MBC, and (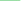) FBC; (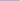) MBC, and (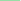) FBC. (**b**) Luminal B group (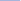) MBC, and (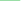) FBC; (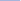) MBC, and (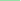) FBC.

Further, we grouped the patients by molecular subtype according to ER, PR, and HER2 expressions and extensively compared the outcomes between MBC patients and FBC patients. Since the numbers of TNBC and HER2‐positive patients in the MBC group were extremely rare, we defined the two types as the same group and named it the nonluminal subtype. Interestingly, similar OS and DFS were found between the MBC and FBC groups (log‐rank *P* > 0.05) for the patients in the nonluminal subtype (log‐rank all *P* > 0.05) (Fig 2). However, for the patients in the subgroup of luminal A and luminal B, the MBC prognosis was significantly worse than that of FBC (log‐rank all *P* < 0.05) (Fig 3). Therefore, we could see the biology of MBC patients was not the same as that of FBC patients.

Tables 3 and 4 summarize the results of univariate analysis of DFS and OS for MBCs and FBCs. Patients with tumor size <5 cm (*P* = 0.000) and lymph node negative (*P* = 0.001 for DFS; *P* = 0.000 for OS) were associated with better survival both for MBCs and FBCs. Regarding treatment modalities, radical mastectomy, chemotherapy and hormonal therapy were also significantly associated with increased survival in the two groups (all *P* < 0.05). However, when analyzed by other parameters such as ER, HER‐2 status and molecular subtypes, a statistical association existed with the prognosis of FBC patients but not with that of the MBC patients. Thus, we speculated a different effect of molecular subtypes on MBC prognosis compared with that on FBC patients. We analyzed the prognostic significance of endocrine therapy in 101 ER positive patients, and concluded that endocrine therapy could significantly improve the PFS and OS (*P* = 0.025 for DFS; *P* = 0.034 for OS, shown in Table 3).

**Table 3:** Univariate analysis of overall survival (OS) and disease‐free survival (DFS)

		DFS			OS		
Parameter	No.of cases	Five years (%)	χ^2^	*P*‐value	Five years (%)	χ^2^	*P*‐value
Age			0.189	0.665		0.711	0.399
> 50	82	65.3			78.8		
≤ 50	70	62.8			72.9		
Tumor size			23.125	0.000*		19.325	0.000*
≤ 5 cm	132	70.9			80.3		
> 5 cm	20	20.3			34.6		
Lymph node			11.690	0.001*		12.973	0.000*
Negative	85	73.4			84.7		
Positive	67	45.3			58.5		
ER/PR status			0.065	0.802		0.912	0.332
Negative	20	60.2			76.3		
Positive	101	68.4			72.4		
HER2 status			0.712	0.542		3.003	0.057
Negative	45	69.8			78.5		
Positive	18	64.1			70.9		
Surgery			20.531	0.000*		23.032	0.000*
RM^a^	129	70.6			89.7		
Non‐RM^b^	23	20.5			49.3		
Chemotherapy			4.521	0.030*		6.001	0.021*
Negative	40	52.8			60.5		
Positive	112	65.7			80.3		
Radiotherapy			0.042	0.086		0.778	0.378
Negative	90	59.6			72.9		
Positive	62	61.5			79.1		
Endocrine therapy^c^			8.124	0.025*		5.021	0.034*
Negative	42	50.1			62.3		
Positive	59	69.8			79.6		

^a^ Radical mastectomy.

^b^ Nonradical mastectomy;.

^c^ In 101 ER/PR positive patients.

* *P* < 0.05.

**Table 4:** Univariate analysis of overall survival (OS) and disease‐free survival (DFS)

	DFS		OS	
Variable	χ^2^	*P*‐value	χ^2^	*P*‐value
For MBC				
Age > 50	0.189	0.665	0.7	0.399
Tumor size (>5cm)	23.125	0.000*	19.325	0.000*
Lymph node positive	11.690	0.001*	12.973	0.000*
ER positive	0.065	0.802	0.912	0.332
HER2 positive	0.712	0.542	3.003	0.057
Radical mastectomy	20.531	0.000*	23.032	0.000*
Chemotherapy	4.521	0.030*	6.001	0.021*
Endocrine therapy	10.124	0.006*	5.367	0.026*
Radiotherapy	0.042	0.086	0.778	0.378
Luminal‐like subtype	0.869	0.351	2.969	0.085
HER‐2 subtype	0.870	0.351	0.341	0.559
TNBC	0.056	0.986	0.125	0.900
For FBC				
Age > 50	2.456	0.293	3.167	0.088
Tumor size (>5 cm)	8.178	0.006*	15.748	0.000*
Lymph node positive	6.150	0.020*	8.265	0.012*
ER positive	6.183	0.045*	9.541	0.032*
HER2 positive	6.691	0.014*	10.301	0.002*
Radical mastectomy	0.354	0.708	0.790	0.409
Chemotherapy	5.280	0.022*	7.248	0.012*
Endocrine therapy	4.00	0.046*	6.247	0.019*
Radiotherapy	0.484	0.586	0.781	0.677
Luminal‐like subtype	5.073	0.033*	9.073	0.004*
HER‐2 subtype	4.889	0.044*	5.723	0.036*
TNBC	5.235	0.047*	4.209	0.038*

* *P* < 0.05.

As shown in Table 5, in the multivariate analysis, tumor size, surgery modalities and endocrine therapy remained independent prognostic factors for DFS and OS of MBCs (all *P* < 0.05).

**Table 5:** Multivariate analysis of overall survival (OS) and disease‐free survival (DFS)

	DFS		OS	
Variable	HR (95% CI)	*P*‐value	HR (95% CI)	*P*‐value
For MBC				
Tumor size (>5 cm)	3.284 (1.327–8.130)	0.010*	2.422 (1.021–5.748)	0.045*
Lymph node positive	1.685 (0.931–3.047)	0.085	1.874 (1.078–3.258)	0.026*
Radical mastectomy	0.275 (0.126–0.599)	0.001*	0.218 (0.096–0.497)	0.001*
Chemotherapy	0.937 (0.385–2.280)	0.886	1.175 (0.493–2.803)	0.716
Endocrine therapy	1.718 (1.022–2.888)	0.041*	1.844 (1.095–3.107)	0.021*
For FBC				
Tumor size (>5 cm)	4.291 (1.182–15.578)	0.027	5.328 (1.537–18.463)	0.008
Lymph node positive	1.009 (0.304–3.293)	0.859	1.431 (0.587–3.490)	0.431
ER positive	0.945 (0.659–1.355)	0.759	0.834 (0.629–1.105)	0.206
HER2 positive	1.218 (0.656–3.336)	0.048*	2.320 (1.264–4.557)	0.026*
Chemotherapy	2.513 (1.357–4.655)	0.003*	2.972 (1.600–5.524)	0.001*
Endocrine therapy	2.584 (1.338–4.992)	0.004*	4.814 (2.097–11.050)	0.002*
Radical mastectomy	1.009 (0.304–3.293)	0.859	1.431 (0.587–3.490)	0.431
Luminal‐like subtype	1.613 (1.002–2.597)	0.049*	1.590 (1.012–2.500)	0.044*
HER‐2 subtype	1.720 (1.068–3.561)	0.037*	2.320 (1.264–4.557)	0.026*
TNBC	0.527 (0.378–0.736)	0.056	0.480 (0.291–0.645)	0.053

* *P* < 0.05.

## Discussion

Breast cancer is the most common malignancy among females worldwide, including China.^15^, ^16^ Compared to FBC patients, the incidence of breast cancer in men is lower, but its incidence has been shown to have increased by about 26% in the past two decades.^17^ MBC patients contribute 1% to 1.2% of all breast cancers in Western countries.^1^, ^7^, ^8^ According to the reports from Korea and Japan, the incidence of MBC is low (0.48 and 0.5%),^18^, ^19^ but the data from China remains limited. In our cohort, the incidence of MBC was 0.62%, which is similar to that in East Asian countries. Our series was based on a retrospective analysis of MBC patients treated in the same center. Mainly in consonance with other studies,^7^, ^20^ our data suggest MBC patients tend to present with larger tumors and at a higher nodal stage, which may be related to lack of male mammogram screening and also less awareness of breast cancer. Additionally, MBC patients often present with a mass centrally located which has usually invaded the nipple as previously reported.^1^

In Western countries, MBC has been reported to occur more often in older men with a median age at diagnosis in the mid‐60s. In Korea, MBC was also diagnosed nearly 10 years later than FBC with a median age of 63‐years‐old.^18^ In our study, we obtained an opposite result with a median age of 58 years in MBC patients, which was similar to that of FBC patients but younger than that reported in all foreign studies. This might be a unique tumor biology in Chinese men. A family history of breast and ovarian cancer has been reported in approximately 15%–20% of MBC patients, conferring a relative risk of 2.5.^3^, ^21^ We also found a significantly higher rate of cancer family history in the MBC group than in the FBC group. The familial aggregation of cancer may provide evidence of the genomic background of MBC morbidity. Therefore, The American Society of Clinical Oncology (ASCO) recommends that all MBC should be offered genetic counseling and testing, regardless of family history.^3^

ER positivity has been reported in 80%–100% of MBC patients^8^, ^18^, ^22^ which is confirmed as higher than that in FBC patients. In our study, the rate of ER positivity was 66% in the MBC group and 49% in the FBC group, which was lower than that reported in previous studies. The difference might be due to a stricter standard for definition of ER positivity used in our cohort (15% as a positive cutoff value). ER positivity was definitely associated with better prognosis in FBC patients, but our observation was that ER‐positive status was not found to be prognostic, perhaps owing to the fact that our ER‐negative cohort included patients with low ER expression according to the 2013 St Gallen consensus guidelines. HER2 is a transmembrane receptor protein which is overexpressed in approximately 35% of FBC and associated with a worse prognosis.^23^ In contrast, HER‐2 overexpression is uncommon in MBC patients. Bloom *et al*. performed a large‐scale research and found HER2 positivity was only 1.7% in the MBC patients compared with 26% in the FBC patients.^24^ In the current series, HER2 positivity was observed in 11.3% of the MBC group, which was higher than that in Western reports. However, although a prognostic marker in FBC patients, no significant association between HER‐2 status and prognosis was seen in our MBC group, which may be due to the small number of HER2‐positive patients.

Previous reports on molecular subtyping and its value on estimating prognosis of MBC tumors are scarce. Abreu *et al*. used an IHC panel of ER, PR, HER2, and ki67 on 111 MBCs to delineate subgroups.^5^ They observed that most cases (89%) were luminal A, 7% luminal B, and only 4% TNBC and <1% HER2 phenotype. As observed in previous reports, luminal A phenotype was the most frequent subtype, and TNBC subtype was rarely seen in the present study. Compared with luminal A and luminal B subtype in the MBC group, We did not identify the nonluminal type (including TNBC and HER2) as the aggressive subtype, which was possibly associated with the rare numbers of HER2 and TNBC subtype in our MBC series. As revealed in several previous studies,^25^, ^26^, ^27^ FBC subtypes do not give the same prognostic information in MBC patients, even in luminal A/B groups. We found that in patients with luminal A/B subtypes, MBC patients had a worse prognosis than FBC patients. MBC has been demonstrated to be a disease distinct from FBC.^26^, ^28^Therefore, new subgroups are warranted to better understand tumor behavior and provide optimal management for MBC patients.^5^, ^29^

Over the past decades, there has been great progress in the local and systemic management of FBCs, but these advances have rarely been applied to the management of MBC. We observed that most MBC patients underwent modified radical mastectomy which has been previously reported in many studies.^30^ The rate of breast conserving surgery (BCS) is still low in China for educational and economic reasons. Thus, in our cohort, the proportion of BCS was very low in both MBC and FBC patients, and an even lower BCS rate was found in the MBC group. The difference might be partially due to most MBC patients presenting with a centrally located mass involving the nipple. However, recent studies have suggested that compared with modified radical mastectomy, breast conserving surgery is a safe and effective option in select MBC patients. Therefore, less aggressive surgical approaches, such as BCS as well as sentinel lymph node biopsy, are recommended in MBC patients.^8^, ^30^ With regard to adjuvant treatments, the rate of endocrine therapy was performed less in the MBC group, despite more MBC patients presenting with ER‐positive breast cancers. However, we firmly accept that endocrine therapy is of great importance in the treatment of ER‐positive patients owing to its significant association with better prognosis in our cohorts, which has also been reported in other studies.^8^, ^31^ Tamoxifen is recommended over aromatase inhibitors in endocrine therapy for MBC patients, as it is unclear if aromatase inhibitors adequately reduce estrogen levels in MBCs.^31^, ^32^ Therefore, all MBC patients in our study received tamoxifen treatment except one patient who received an aromatase inhibitor. Chemotherapy and radiation therapy were administered in 73.7% and 40.8% in MBC patients. In accordance with previous studies,^7^, ^8^ we also observed that chemotherapy was associated with a better DFS and OS in male patients.

Data regarding the specific prognosis of MBC patients in the literature are conflicting, whereas more recent studies agree with a similar prognosis to that of FBC.^1^, ^8^, ^20^ Based on a Chinese population with long‐term follow‐up, our study showed worse OS and DFS in MBC patients than in FBC patients. Multivariate analysis determined that in the whole population including MBC and FBC patients, endocrine therapy was an independent risk factor for OS and DFS. As for MBC patients, radical mastectomy was another adverse prognostic factor. In addition to treatment‐related variables, we also identified several tumor‐related parameters such as higher T and N classifications associated with a worse prognosis in MBC patients. Therefore, the poor prognosis of MBC patients in our sample was possibly associated with both aggressive tumor characteristics and inadequate treatment models. We believe that in the treatment of MBC patients, radical surgery should be pursued to improve their prognosis; in addition, for ER + MBC patients, endocrine therapy should not be ignored as it can greatly improve their prognosis.

In conclusion, our data show that compared with FBC patients, MBC patients showed a higher rate of lymph node invasion, ER positivity and HER2 negativity. Additionally, luminal A type was the most common subtype in MBC patients, yet TNBC and HER2‐positive subtype were rare. Remarkably, MBC patients showed a worse outcome compared with FBC patients. The poor prognosis of MBC patients correlated with both different biological characteristics and inadequate treatment models in MBC patients.

## Disclosure

No authors report any conflict of interest.
